# Clear cell “sugar” tumor of the lung: Diagnostic characteristics of a rare pulmonary tumor: A case report and review of literature

**DOI:** 10.1097/MD.0000000000033035

**Published:** 2023-02-17

**Authors:** Bo Wang, Xin Xu, Zhenya Zhao

**Affiliations:** a Department of Pathology, Xingtai People’s Hospital Affiliated to Hebei Medical University, Xingtai, Hebei, P.R. China.

**Keywords:** clear cell tumor, glycogen, lung mass, PEComa, sugar tumor

## Abstract

Clear cell tumors of the lung (CCTLs), also known as “sugar tumors” for an abundant cellular glycogen concentration, are an extremely rare type pulmonary neoplasm. Often, they are incidentally found on chest roentgenogram or computed tomography scan during routine examination. CCTLs usually present with nonspecific symptoms that pose a diagnostic challenge to clinicians. Accordingly, histopathology remains the gold standard for diagnosing. Moreover, some of them can present with either appearances or histopathological features similar to other pulmonary neoplasms under the light microscope, including pulmonary malignancy, thereby causing misdiagnosis prior to or after surgery. Accordingly, herein, we describe a rare case of CCTL, review the literature has been published, and then discuss the benign versus malignant nature of this rare tumor.

**Patient concerns:**

A 59-year-old man presented due to a high-density chest nodule in the left diaphragm. The patient’s medical history was unremarkable and he also denied smoking in the past.

**Diagnosis:**

Physical examination, there were no noted signs. A new chest contrast-enhanced computed tomography revealed a 3.2 × 2.5 cm, solitary, circular nodule with a smooth edge located in the beside of the left thoracic aorta. Postoperative pathological and immunohistochemical examinations of the surgical specimens revealed a final diagnosis of CCTLs.

**Interventions:**

The patient underwent video-assisted thoracoscopic surgery. A wedge resection of left lower lung lobe was carried out and the tumor node was successfully removed alongside normal surrounding parenchyma.

**Outcomes:**

The operation was successful. Then the patient recovered completely and continued to do well on postsurgical thoracic surgical clinic visits. The tumor was a benign tumor, and the patient did not require any additional treatment. The patient had been followed-up regularly for 4 years after surgery; she did not experience any complications and remained disease-free.

**Conclusion:**

CCTLs should be considered in the differential diagnosis if a patient shows a solitary, circular chest nodule with a smooth edge. They are extremely rare lung tumors that must be differentiated from other lung tumors, especially the malignant tumors. Although pathological and immunohistochemical findings are important for making the diagnosis, the varying histopathological features on microscope make diagnosis difficult. The current case highlights the importance of physicians being aware of and suspecting CCTLs in similar cases, along with knowing the characteristics of CCTLs for the diagnosis and differential diagnosis.

## 1. Introduction

Clear cell tumors of the lung (CCTLs) are extremely rare primary pulmonary lesions that were first described by Liebow and Castleman in 1963,^[1]^ and since then only approximately 60 cases have been reported in the literature. This type of pulmonary tumor consists of clear cells that have high levels of glycogen and thin cell walls, thus is otherwise called “sugar tumor” and showing extensive positivity for periodic acid-Schiff staining. According to the 2015 World Health Organization (WHO) classification, CCTLs are benign tumors, belonging to the family arising from putative perivascular epithelioid cells, and are named as “PEComatous tumors” of the lung.^[2]^ These tumors generally have a benign prognosis, although a few cases with multiple nodules or a malignant course have been reported.^[3,4]^ Accordingly, The definite diagnosis is still dependent on the pathological morphology and immunohistochemical examination. Moreover, some of CCTLs can present with either appearances or histopathological features similar to other pulmonary neoplasms, and the pathologic morphology is easily confused with other similar pathological changes such as metastatic renal clear cell carcinomas in the lungs, thereby causing misdiagnosis prior to or after surgery. Accordingly, it is important to be familiar with features of diagnosis and differential diagnosis of CCTLs, which can help to make the correct diagnosis; awareness of this entity, familiarity with the associated clinical features and recognition of the cytomorphologic features of CCTLs all can help doctors avoid certain pitfalls in the diagnostic and treatment process. Accordingly, herein, we present a case of CCTL occurring in a 59-year-old male patient, and review the literature has been published. The descriptions of CCTLs in this case and the review features of CCTLs, based on published cases, should add to pathologists knowledge of diagnosis, and then assist them with diagnosis; they also can help clinicians improve the understanding of this rare disease, and futher avoid certain overtreatment. Meanwhile, the benign versus malignant nature of this rare tumor will be discussed in this report.

## 2. Case report

**Figure 1.**
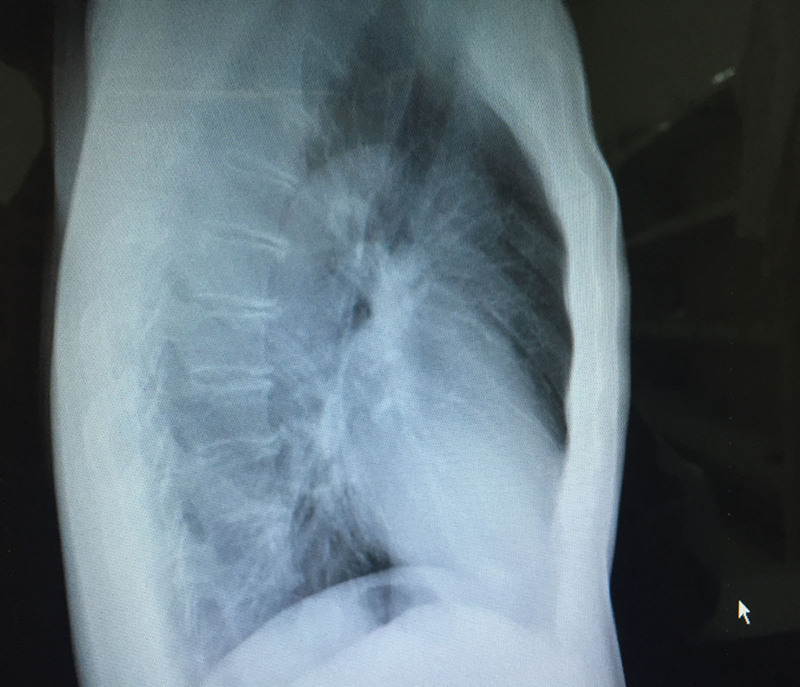
Chest X-ray displaying the high-density nodule in the medial side of the left diaphragm.

A 59-year-old man was referred to our hospital for further treatment due to a high-density chest nodule in the left diaphragm, which was accidentally found on chest roentgenogram during routine examination in other hospital (Fig. 1). The patient’s medical history was unremarkable and he also denied smoking in the past. This case was treated in accordance with the ethical standards of Xingtai People’s Hospital.

**Figure 2.**
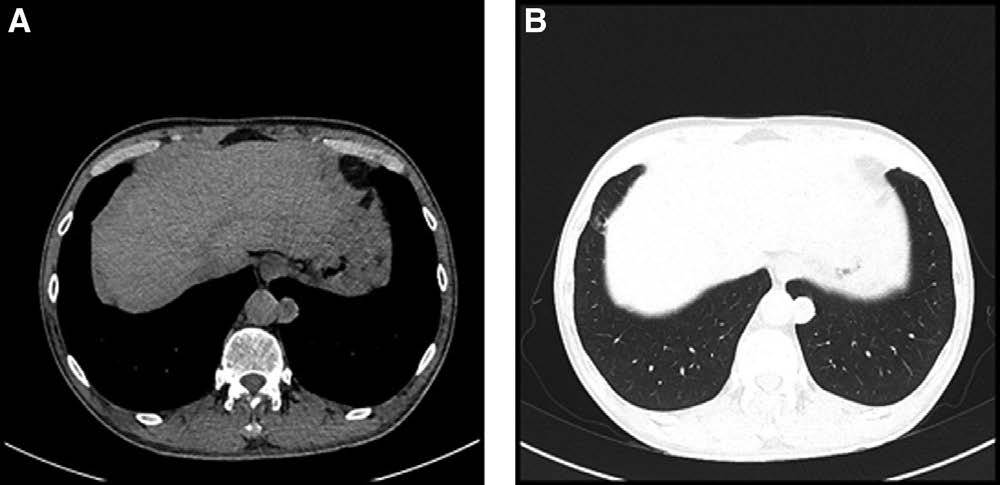
Computed tomography (CT) features of CCTL. (A, B) Contrast-enhanced CT tomography thorax-axial and coronal section showing the mass. (C, D) Contrast-enhanced CT tomography coronal section showing the mass. CCTLs = clear cell tumors of the lung, CT = computed tomography.

Physical examination, there were no noted signs of cough, hemoptysis, shortness of breath or evidence of voice hoarseness. A new chest contrast-enhanced computed tomography (CT) revealed a 3.2 × 2.5 cm, solitary, circular nodule with a smooth edge located in the beside of the left thoracic aorta and was flush with the thoracic 12 vertebral body. CT chest scan also showed with unclear boundary between the nodule and left thoracic aorta, but without mediastinal lymph node enlargement (Fig. 2A and B). The while fiberoptic bronchoscopy showed neither stenosis of bronchi nor any endobronchial mass. The patient underwent video-assisted thoracoscopic surgery, and a 3 cm subpleural lesion without pleural abnormality, located in the posterior basal segment of the left lower lobe of lung, was visualized. A wedge resection of left lower lung lobe was carried out and the tumor node was successfully removed alongside normal surrounding parenchyma. The rest of the lung appeared normal with no pleural effusions or any other abnormalities. Frozen sections of the tumor node showed no evidence of malignancy. The operation was successful. Then the patient recovered completely and continued to do well on postsurgical thoracic surgical clinic visits.

**Figure 3.**
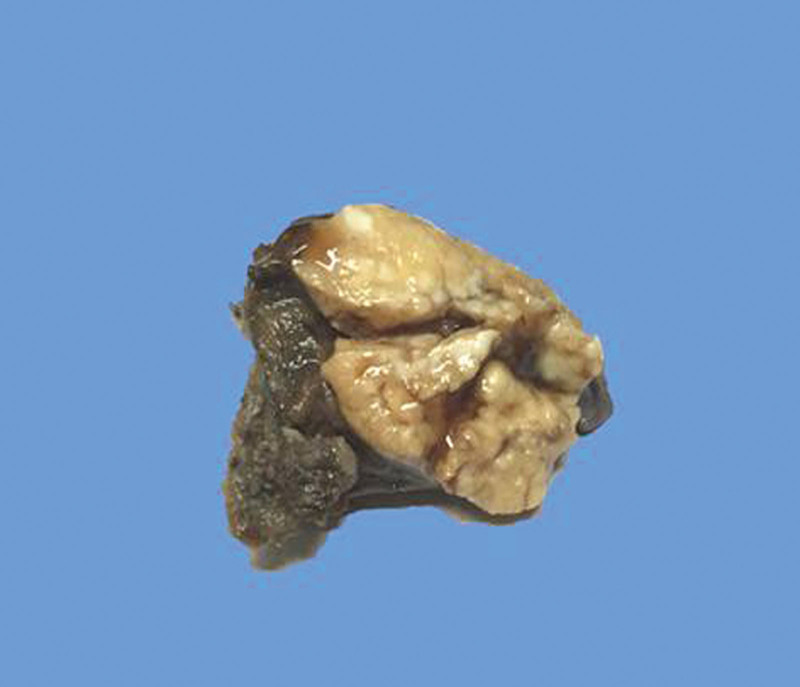
Resected left lower lobe of lung sample showing a well-circumscribed, solid, creamy white tumor mass measuring 3.0 cm in greatest diameter.

The resected specimen was sent for histopathologic examination. On gross examination, a solid well-circumscribed mass was found in the parenchyma of the resected lung tissue. The mass measured 3 × 2.5 × 2 cm, presented a homogenous creamy white appearance on inspection, and had not a fibrous capsule (Fig. 3).

**Figure 4.**
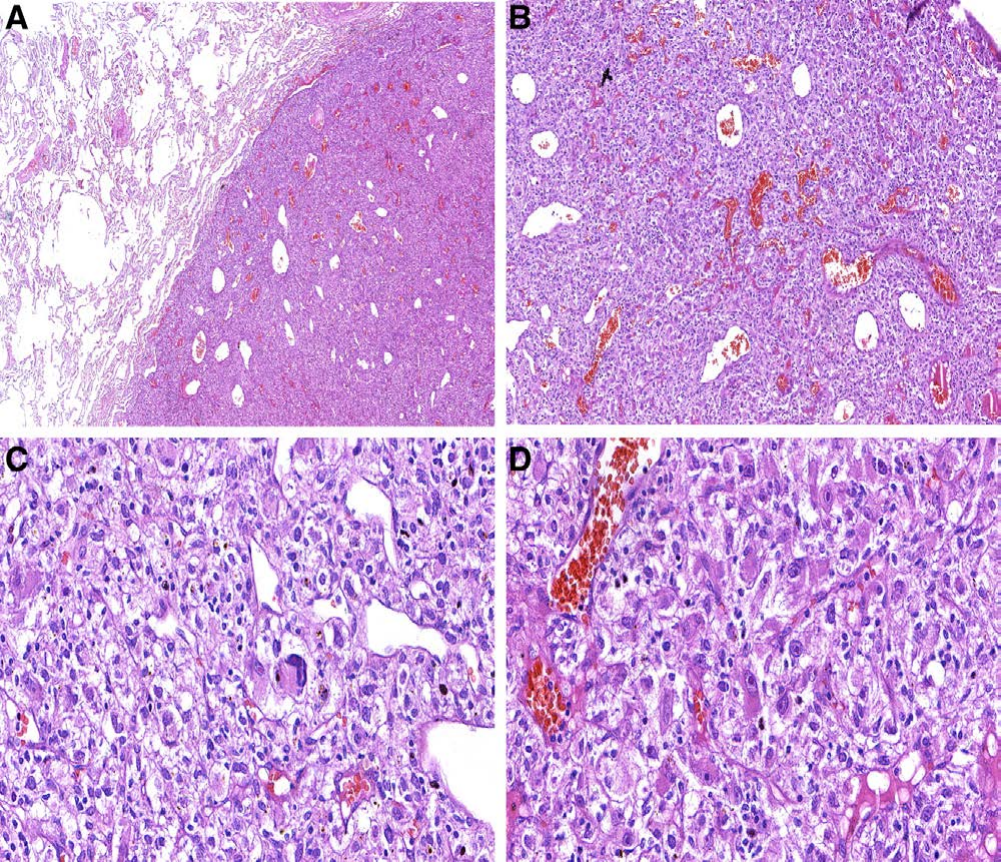
Histological characteristics of CCTL. (A) The tumor does not have a fibrous capsule. Magnification, ×40. (B) The tumor presents with a solid cellular growth pattern, and the neoplastic cells are separated by a rich delicate capillary network and sinusoid-like vessels. Magnification, ×100. (C, D) On higher magnification, these neoplastic cells are arranged around the vessels; they appearing epithelioid-like with round or ovoid, polymorphic nuclei, abundant clear to pale eosinophilic cytoplasm and inconspicuous mitotic activity. Magnification, ×400. CCTLs = clear cell tumors of the lung.

Light microscopy examination showed that the mass did not have a fibrous capsule (Fig. 4A). Microscopy of the mass revealed a proliferation of round and oval cells arranged in insular or sheeted patterns; some neoplastic cells also are arranged around the vessels; the neoplastic cells were separated by a rich delicate capillary network and sinusoid-like vessels (Fig. 4B). They were large ones with round to oval, polymorphic nuclei, indistinct to apparent nucleoli and abundant clear to pale eosinophilic cytoplasm; they all had a low nuclear to cytoplasmic ratio and lacked mitotic activity. Meanwhile, no necrosis, hemorrhage or cyst formation was observed in the mass (Fig. 4C and D).

**Figure 5.**
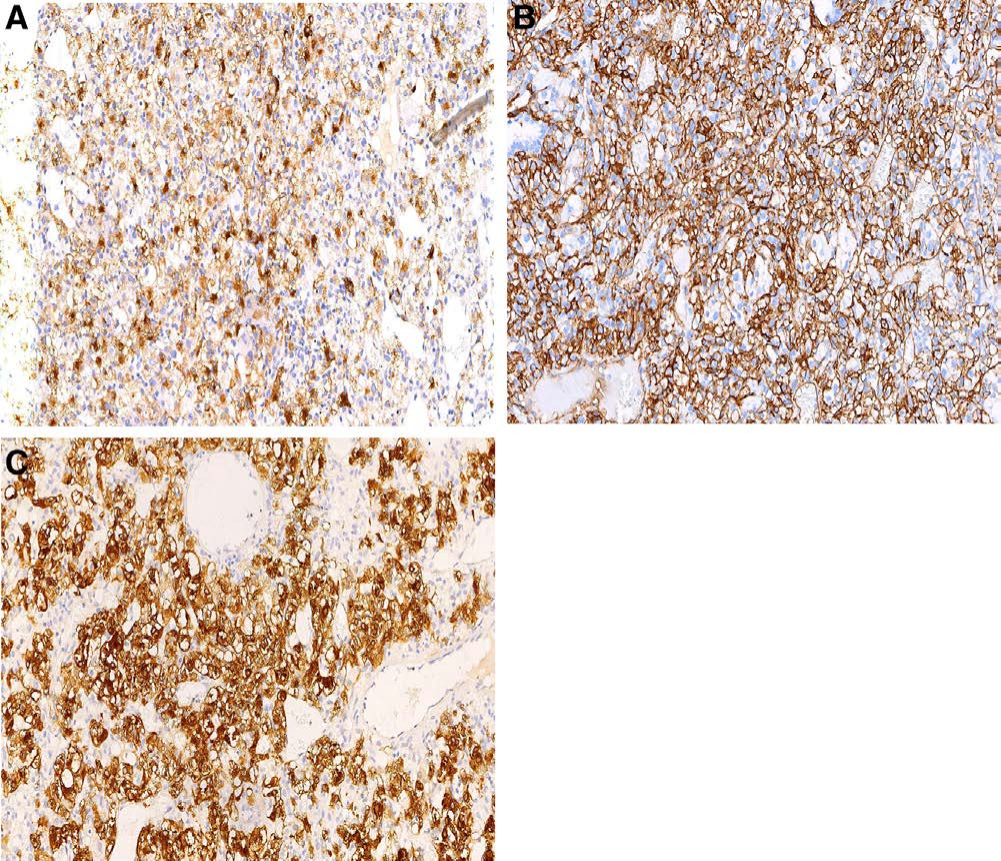
Immunohistochemistry staining of the CCTL. The tumor cells stained positive for (A) HMB-45. Magnification, ×200. (B) CD34 and (C) Melan A. Magnification, ×200. CCTLs = clear cell tumors of the lung.

Immunochemical analysis displayed the tumor cells were diffusely positive for HMB45 (Fig. 5A) and CD34 (Fig. 5B), patchily stained for Melan-A (Fig. 5C); they were negative for Vimentin, CD1a, pan cytokeratin cocktail AE1/AE3, cytokeratin 7, cytokeratin 20, epithelial membrane antigen (EMA), thyroid transcription factor-1, smooth muscle actin (SMA), synaptophysin (Syn), chromogranin (CgA) and S-100. The Ki-67 index was approximately 3% to 5%. Thus, the findings supported a final diagnosis of clear cell tumor of the lung (CCTL). The patient has been followed-up regularly and has remained asymptomatic for 2 years subsequent to surgery.

## 3. Discussion

Clear cell tumors or otherwise defined as “sugar tumors” of the lung are extremely rare. Since Liebow and Castleman original report in 1963, only approximately 60 cases of CCTL have been reported in the literature to date. The histogenesis of CCTL remains uncertain. The histological, ultrastructural and immunophenotype of CCTL were characterized by the presence of perivascular epithelial cell (PEC), which was introduced by Bonetti et al^[5]^ in 1992 as an unusual distinctive cell type presented in angiomyolipoma and CCTLs. The PEC had features of epithelioid appearance, clear to eosinophilic cytoplasm with perivascular distribution, surrounding by thin-walled vascular spaces and sinusoid-type vessels, all of the above support a myoid/pericytic origin. Immunohistochemical analysis indicated that the PEC is strongly positive for HMB-45, can positive for Melan-A also focal with S100 and muscle-specific actin.^[5,6]^ Besides, a group of lesions, including angiomyolipoma, lymphangiomyoma, lymphangioleiomyomatosis (LAM), renal capsuloma, and clear cell myelomelanocytic tumor of the ligamentum teres were found to share similar histological, ultrastructural and immunohistochemical features, so be also characterized by the presence of the distinctive cell type of PEC. This group of lesions also has been designated as PEComas.^[7–9]^ To sum up, in the 2015 WHO classification, CCLTs are designated as a number of PEComatous tumors.^[2]^ So far, the specific risk factors for CCTL have not been established.

Between the two genders, there is a slight female preponderance among the CCTL patients.^[10,11]^ Although the age range of the patients have been reported from 8 to 63 years old with the median age of 57 years; the neoplasm seems to present with a higher incidence in the elderly.^[12]^ Usually, the presentation is an asymptomatic patient with an isolated coin lesion, so the lesion often is discovered inadvertently by via radiologic. CCTLs also can cause nonspecific symptoms in a few cases such as coughing, shortness of breath, hemoptysis and so on. Over a long-term observation and research, some unique presentations have been reported in literatures, such as the finding about concurrently with minute pulmonary meningothelial-like nodules in the patient with rectal adenocarcinoma,^[13]^ or concurrently with thrombocytosis (platelet count > 1,000,000/mm³), which was solved after removing the CCTL.^[11,14]^

Radiographically, CCTLs generally present as solitary, round, peripheral and parenchymal nodules with smooth boundary, but without evidence of cavitation or calcification. The location of the tumor has no lobar preference.^[15]^ A recent review showed that CCTLs tended to affect bilateral lower lung fields although they could occur in any lung lobes.^[10]^ On contrast-enhanced CT scans, these lesions might show intense postcontrast enhancement because of their rich vascular stroma. This imaging performance is similar to a malignant neoplasm and thus easily leads to misdiagnosis.

Most of CCTLs are diagnosed by resection of the tumor and histopathology of the resected specimen; however, a few cases have been able to be diagnosed with preoperative transbronchial biopsy or fine needle aspiration biopsy.^[16–18]^ Macroscopically, most CCTLs appear as well-circumscribed, solitary and pink “coin lesions” with diameters generally < 3 cm (ranging from 1–45 cm), but with no necrosis, bleeding and surface fibrous capsule. They are well demarcated from the lung parenchyma. In a few cases, CCTLs can with capsule, appear as large nodules (diameter > 10 cm) or in the bronchus. The light microscopic examination revealed that CCTLs are much more cellular. The neoplastic cells arrang in insular patterns, are separated by a rich delicate capillary network and sinusoid-like vessels (Fig. 2A). CCTL cells are epithelioid-like, moderately and large, with features of similar size, round, oval or polygynous shape; they have small nuclei, small central nucleoli, uniform chromatin, abundant clear to eosinophilic cytoplasm and distinct cell borders. Scanty intervening stroma with prominent thin-walled sinusoidal vessels is also a characteristic in clinicopathology.

The immunohistochemical expression pattern of CCTLs is unique, as well immunoreactivity is a definitive method of diagnosing this special type of tumor. The tumor cells were stable positive for HMB-45, inconstant positive for Melan A, S100, neuron-specific enolase (NSE), Syn, SMA, tyrosinase and microphthalmia transcription factor; while negative for pan cytokeratin cocktail AE1/AE3, EMA, CgA, thyroid transcription factor-1, and so on.^[19,20]^ It has been reported that the most sensitive markers are HMB-45, melan-A, and microphthalmia transcription factor ^[21]^; also, the HEM-45 positive might be an idiosyncratic reaction.^[20]^ The expression of CD34 is strong and diffusely distributed in CCTLs in spite of, it is usually absent or scattered in PEComas that occur in other organs. This finding indicates that the positive staining of CD34 may be important for the diagnosis of CCTLs.^[22]^ Adachi et al^[23^ reported a case of CCTL positively stained for CD1a, and also confirmed CD1a protein positive expression in 19 cases of PEComa.^[24]^ These results suggested that CD1a antibody could be an additional marker for diagnosing PEComas.

Most of them have been considered benign prognosis due to there was no firm evidence for reoccurrence, invasion, or metastasis, so CCLTs are benign tumors according to the latest WHO classification.^[2]^ However, certain malignant cases have been reported in the literature so far, and one case described the death of a patient from metastatic CCTL.^[3]^ The question about the CCTL’s malignancy provokes some interesting discussions, and there have been some literatures had summed up the established associated criteria for the diagnosis of malignant potential CCTLs, which included any combination of the following: infiltrative growth, marked hypercellularity nuclear enlargement and hyperchromasia, high mitotic activity, atypical mitotic figures, coagulative necrosis, larger diamrter (>2.5 cm) with the presence of uncommon symptoms. A recent review revealed that the tumor size is closely related to the patient’s clinical presentation, once the maximum diameter of tumor > 2.2 cm tend to produce symptoms more common than compared with the smaller ones.^[10]^ Furthermore, nodules presented with pure ground-glass opacities in PET images were more likely to be malignant, and the malignant rate was about 59% to 73%.^[25]^

CCTLs should be distinguished from other primary or metastatic tumors of the lung, particularly those with remarkable clear cells, due to these different types of tumor may display similar histological features. Immunohistochemistry can definitively help differentiate CCTLs from other pulmonary masses. The principal tumor typles to exclude in the differential diagnosis are:

Clear cell pulmonary carcinoma and clear cell variants of primary pulmonary carcinoma: These tumors show abundant mitosis, necrosis, and immunoreactivity for cytokeratin, with no reactivity for melanin markers.

Clear cell carcinoid of lung: These clear cancer cells are diffuse positive for Syn, CgA, NSE, cytokeratin, and so on, as well as negative for HMB-45, Malen-A, CD34, and so on. But things areopposite in the CCTL.

Metastatic clear cell carcinoma of the lung: These metastases show differant immunohistochemical expression pattern from CCTLs, for example: Metastases of renal clear cell carcinoma show immunoreactivity for cytokeratin, EMA, CD10 as well as Vimentin, with no reactivity for HMB-45, and Melan-A; Metastases of liver clear cell carcinoma with the presence of immunoreactivity for cytokeratin 8, cytokeratin 18, Hepatocye and EMA, with no reactivity for HMB-45 and Melan-A and CD34; Metastases of ovarian clear cell carcinoma are positive for cytokeratin, ovarian cancer anyigen and EMA, but negative for HMB-45; Metastases of thyroidal clear cell carcinoma are positive for thyrglobulin and negative for HMB-45.

Paraganglioma: it occurs as a functional or nonfunctional lesion with blood vessels inside the tumor. Hence it can show different CT contrast enhancement according to the density of blood vessels. The tumor cells are positive for neuroendocrine markers, such as Syn, CgA and NSE; nevertheless, they are negative for HMB-45, Malen-A, CD34. In addition, the special staining periodic acid-Schiff also is negative.

Clear cell sarcoma or metastatic melanoma: These masses are generally positive for HMB-45, Malen-A along with S100, as well as a higher Ki67 index; they universally have the significant malignant biological behavior. These patients usually have medical histories. However, CCTLs are positive for HMB-45, Malen-A, generally positive for CD34, but negative for S100, with a low Ki67 index. The CCTL patient has no the ralated medical history. Meanwhile, the majority of CCTLs follow a benign course.^[26]^

Granular cell carcinoma: this type of tumor is even rarer and own more abundant cytoplasm. Immunohistochemistry showed that it is positive for S100 but negative for HMB45 and CD34.

It is difficult to differentiate the exact nature of CCTL, because although most of cases have been reported to be benign, there exists a few reports about the CCTL is associated with malignancy, histologically exhibiting lymph vascular invasion, necrosis, and pleomorphism.^[26]^ In view of the difficulty in diagnosing and predicting the biological behavior of CCTL, pathology and immunoreactivity are now the only accurate methods of diagnosing this kind of tumor. So far, the best course of action is surgical resection of the lesion, which is both diagnostic and curative.^[15]^ According to the WHO guidelines, Complete surgical excision is the treatment of choice for CCTLs and is typically sufficient; there is no adjuvant therapy is normally recommended. A few cases of malignant CCTL have been reported in the literature. Therefore it is imperative to schedule follow-ups for the patients to rule out metastasis. In this case, necrosis, calcification, and amitotic index were not found, but the tumor was 3 cm in diameter and the Ki-67 labeling index was 3% to 5%, which suggested malignant potential. Thus, a careful long-term follow-up of this patient is recommended. The patient was disease-free survival after 4-years of follow-up.

In conclusion, our report describes a case of CCTL and comprehensively reviews of the related literature, in order to help pathologists and clinicians with recognizing, diagnosis, differential diagnosis, avoiding misdiagnosis, and instituting therapeutic strategies, besides, to futher shed light on the behavior and outcome of this tumor. The goals are to inform doctors of this rare masses, and to improve the diagnosis and treatment of this rare masses. Because other malignant tumors require an active treatment regimen, a better understanding of the pathological, immunophenotype features, natural history of this rare tumor, and then make a more accurate diagnosis is extremely important. Positive staining of melanin marker, CD34, CD1a, and SMA are helpful in diagnosing this tumor. The patient of CCTL generally was advised to follow-up.

## Author contributions

**Conceptualization:** Zhenya Zhao.

**Data curation:** Xin Xu.

**Project administration:** Bo Wang.

**Validation:** Xin Xu.

**Writing – original draft:** Bo Wang.
